# Supplementary material for: The mRNA architecture of the translation termination site primes programmed stop codon readthrough events in Drosophila
Source: RNA Biol. 2026 May 6;23(1):1–21. doi: 10.1080/15476286.2026.2667707 (PMC13170385; doi:10.1080/15476286.2026.2667707)
Supplement: Supplemental Material [file KRNB_A_2667707_SM3100.docx]

**
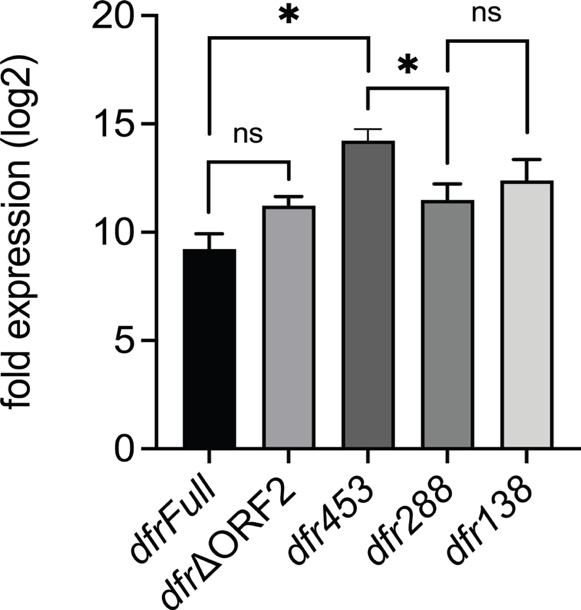
**a b

**
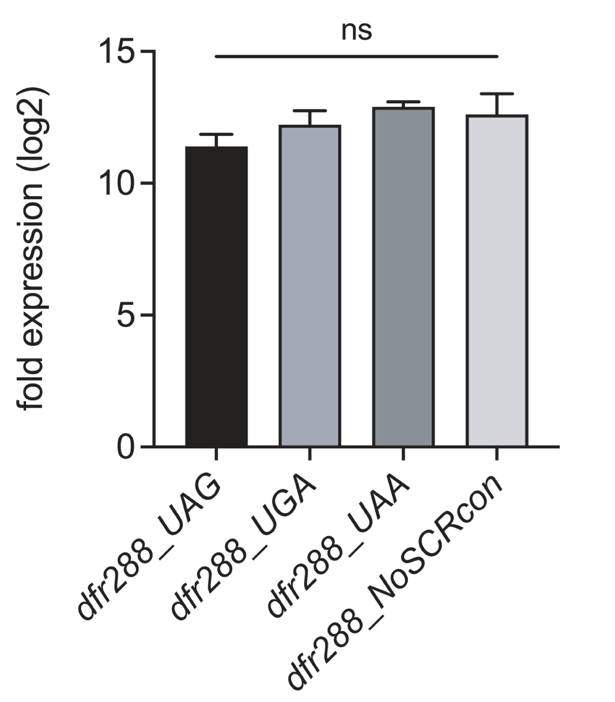
**

**Supplementary Figure S1**

** Supplementary Figure S2**

** Supplementary Figure S3**

**
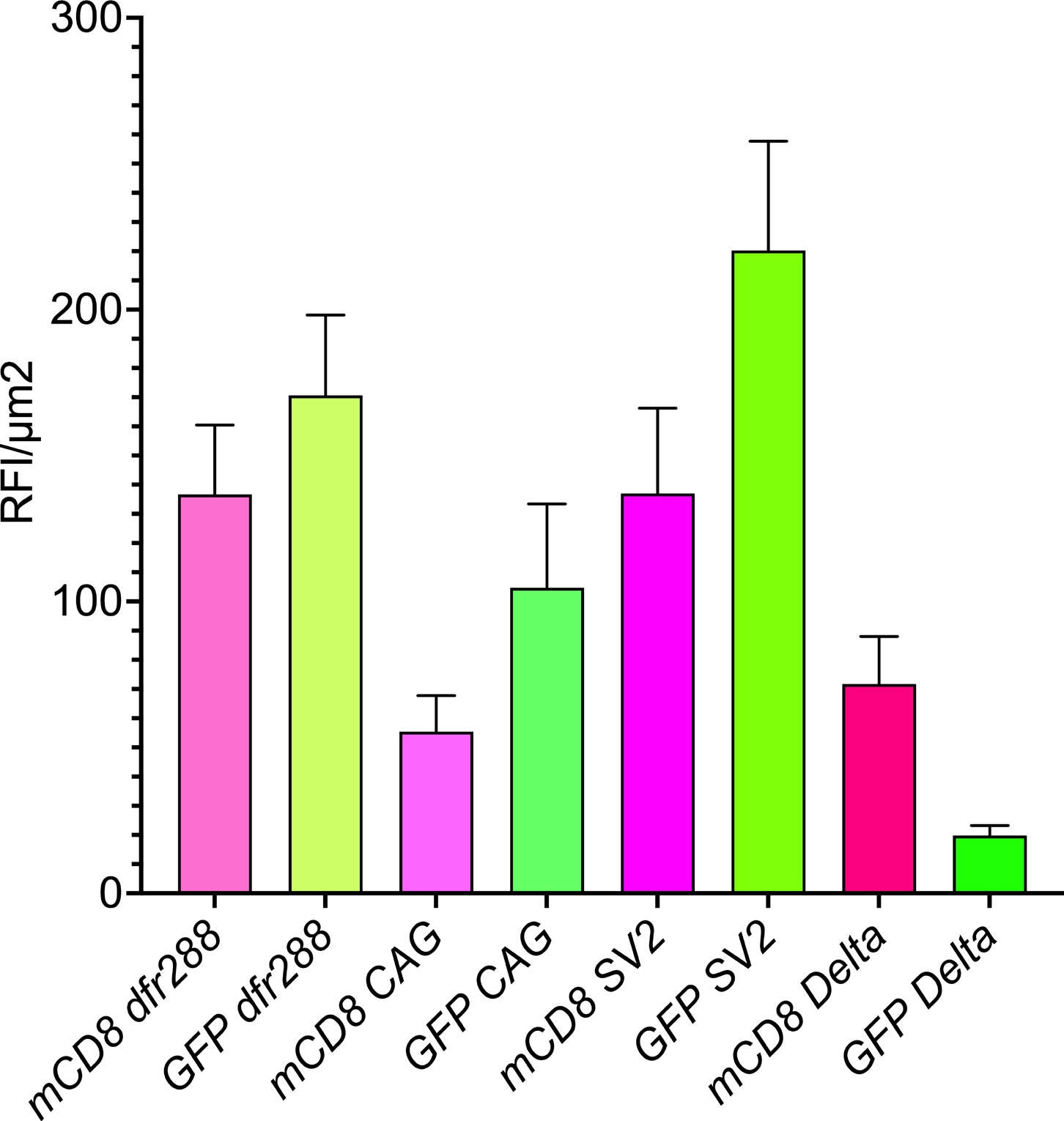
 Supplementary Figure S4**

**Supplementary Figure S5**

**Supplementary Figure Legends**

**Supplementary Figure S1: a and b** Column graphs showing relative expression levels of the reporter transcripts in *Drosophila* S2 cells transfected with the indicated constructs (X-axis), as measured by RT-qPCR. Expression is normalized to *rpl32* mRNA and presented as fold change relative to untransfected cells. Data represent the mean± SEM from three independent biological replicates. Statistical significance was confirmed by one-way ANOVA with Dunnett’s T3 multiple comparisons test (** = p = 0.03,* ns *= p* ≥ *0.05).*

**Supplementary Figure S2:** Evolutionary guide tree inferred from multiple sequence alignment of 29 Drosophila species and two additional out group flies based on their protein orthologs.

**Supplementary Figure S3: a** Schematic redrawing (BioRender.com) of mxFold2-predictions of mRNA secondary structures in the *dfr288*_Stem+3, +6, and +9 sequence variants. The predicted slight variation between the stem-loops results from the algorithm’s priority to select the structure with the lowest MFE. Because of the negligible differences in MFE between all predicted stem-loops (Supp Table ST3), it is more likely that identical stems are formed. **b-e** Column graphs depicting relative expression levels of the reporter transcripts in *Drosophila* S2 cells transfected with the indicated constructs (X-axis), as measured by RT-qPCR. Expression is normalized to *rpl32* mRNA and presented as fold change relative to untransfected cells. Data represent the mean± SEM from three independent biological replicates. Statistical significance was confirmed by one-way ANOVA with Dunnett’s T3 multiple comparisons test (*** = p = 0.002, * = p = 0.03*, ns *= p* ≥ *0.05)*. **f, g** Box plot diagram of the Relative Readthrough Efficiencies (see methods) after transfection of *Drosophila* S2 cells with the respective *dfr_453*-based, destabilizing (DV) (**f**) and stabilizing (SV) (**g**) variants*.* In all box plot diagrams, boxes represent the 25-75% quartile, lines represent the median, whiskers represent min to max. Statistical significance was confirmed by one-way ANOVA with Dunnett’s T3 multiple comparisons test (*n* = 3 independent experimental replicates, **** = p* ≤ *0.001, ** = p = 0.002, * = p = 0.03*).

**Supplementary Figure S4:** Column graph depicting the mean, area-normalized, relative fluorescence intensities (RFI/µm^2^) of mCD8 (pinks) and GFP (greens), revealed by immunofluorescence staining, in PGs of *Drosophila* L3 larvae expressing the indicated *dfr* SCR reporter transgenes under *spok-Gal4.1.45* driver control.

**Supplementary Figure S5: a** Venn diagram depicting sets of genes (mRNAs) with a predicted RNA stem-loop structure in the +80 nt region, downstream of a stop codon (yellow circle), with evidence for SCR obtained from Ribo-Seq data of *Drosophila* embryos (blue circle), and with conserved ORFs downstream of stop codons (green circle). **b** Euler diagram illustrating the intersection among gene sets (mRNAs) identified to have an RNA stem-loop structure within the 3’ UTR with two length parameters, +60 nt *vs* +80 nt. **c** Euler diagram illustrating the overlap between the predicted structures derived from +60 and +80 nt gene sets, which were also demonstrated or predicted to exhibit stop codon readthrough. **d** Column graphs depicting relative expression levels of the reporter transcripts in *Drosophila* S2 cells transfected with the indicated stem-loop variants (X-axis), as measured by RT-qPCR. Data represent the mean± SEM from three independent biological replicates. Statistical significance was confirmed by one-way ANOVA with Dunnett’s T3 multiple comparisons test (*** = p = 0.002,* ns *= p* ≥ *0.05*).
